# Single-cell Transcriptome Mapping Identifies Common and Cell-type Specific Genes Affected by Acute Delta9-tetrahydrocannabinol in Humans

**DOI:** 10.1038/s41598-020-59827-1

**Published:** 2020-02-26

**Authors:** Ying Hu, Mohini Ranganathan, Chang Shu, Xiaoyu Liang, Suhas Ganesh, Awo Osafo-Addo, Chunhua Yan, Xinyu Zhang, Bradley E. Aouizerat, John H. Krystal, Deepak C. D’Souza, Ke Xu

**Affiliations:** 1 Center for Biomedical Information and Information Technology, National Cancer Institute, Rockville, MD 20850 USA; 2 Department of Psychiatry, Yale School of Medicine, 300 George street, Suite 901, New Haven, CT 06511 USA; 3 Connecticut Veteran Healthcare System, West Haven, CT 06516 USA; 4 Bluestone Center for Clinical Research, College of Dentistry, New York University, New York, NY 10010 USA; 5 Department of Oral and Maxillofacial Surgery, College of Dentistry, New York University, New York, NY 10010 USA

**Keywords:** Genetics, Gene expression, Molecular medicine

## Abstract

Delta-9-tetrahydrocannabinol (THC) is known to modulate immune response in peripheral blood cells. The mechanisms of THC’s effects on gene expression in human immune cells remains poorly understood. Combining a within-subject design with single cell transcriptome mapping, we report that THC acutely alters gene expression in 15,973 blood cells. We identified 294 transcriptome-wide significant genes among eight cell types including 69 common genes and 225 cell-type-specific genes affected by THC administration, including those genes involving in immune response, cytokine production, cell proliferation and apoptosis. We revealed distinct transcriptomic sub-clusters affected by THC in major immune cell types where THC perturbed cell-type-specific intracellular gene expression correlations. Gene set enrichment analysis further supports the findings of THC’s common and cell-type-specific effects on immune response and cell toxicity. This comprehensive single-cell transcriptomic profiling provides important insights into THC’s acute effects on immune function that may have important medical implications.

## Introduction

With the increasing rates of cannabis use for recreational and medical purposes, it is important to address a gap in our understanding of its impact on immune and inflammatory functions^1,2^. Delta-9-tetrahydrocannabinol (THC), the principal psychoactive constituent of cannabis, powerfully modulates immune function in peripheral blood cells^3^, in part, through activating cannabinoid receptor 2 (CBR2)^1,3–6^. *In vitro* studies of cannabis exposure, which contains over 450 compounds, show that it modulates immune function^7–10^, changes cytokine production^8,11,12^, inhibits cell proliferation^2^, and induces apoptosis^13,14^. However, little is known about the mechanisms of *in vivo* THC exposure on the transcriptomes of distinct types of peripheral blood mononuclear cells (PBMCs) in humans.

Single cell RNA-seq (scRNA-seq) offers an unprecedented resolution to detect drug effects on cell-specific gene expression^15,16^ and enables the evaluation of molecular aspects of immune cell heterogeneity^17^. Few studies have applied scRNA-seq to detect differentially expressed genes (DEGs) induced by drug exposure, and none have evaluated the effects of THC in humans. This limitation is due mostly to high inter-individual transcriptomic variability and types of cells that confound the assessment of the impact of environmental factors. Most recently, a scRNA-seq study identified a large number of common and cell-type-specific DEGs for Alzheimer disease, suggesting the improvement of analytical methods to overcome the challenge of high transcriptomic variability^18^. Here, we report the first scRNA-seq study using within-subject combined with linear mixed model (LMM) analysis to detect genes affected by intravenous (IV) THC at single cell resolution. It is conceivable that other routes of administration of THC, e.g., pulmonal inhalation or oral ingestion, may affect gene expression differently than IV THC. However, in this first study attempting to analyze the effects of cannabinoids on gene expression in humans, we chose to administer THC intravenously in order to control for a number of potential confounders such as inter- and intra-individual variability in the bioavailability of smoked cannabis or THC^19–22^.

## Results

### Single cell RNA-seq profiling identifies cell types and sub-cell clusters in peripheral blood mononuclear cells (PBMCs)

In this study, samples of blood were drawn and PMBCs were extracted prior to (pre-THC) and 70 minutes following (post-THC) a single 0.03 mg/kg intravenous dose of THC in two healthy individuals. The selected THC dose reliably produces effects consistent with cannabis intoxication^23,24^. The timing of the blood samples was selected to maximize the likelihood of detecting changes in drug-induced gene expression. A battery of subjective and cognitive assessments was administered to capture the effects and safety of THC^23,25,26^ (See Methods).

**Figure 1:**
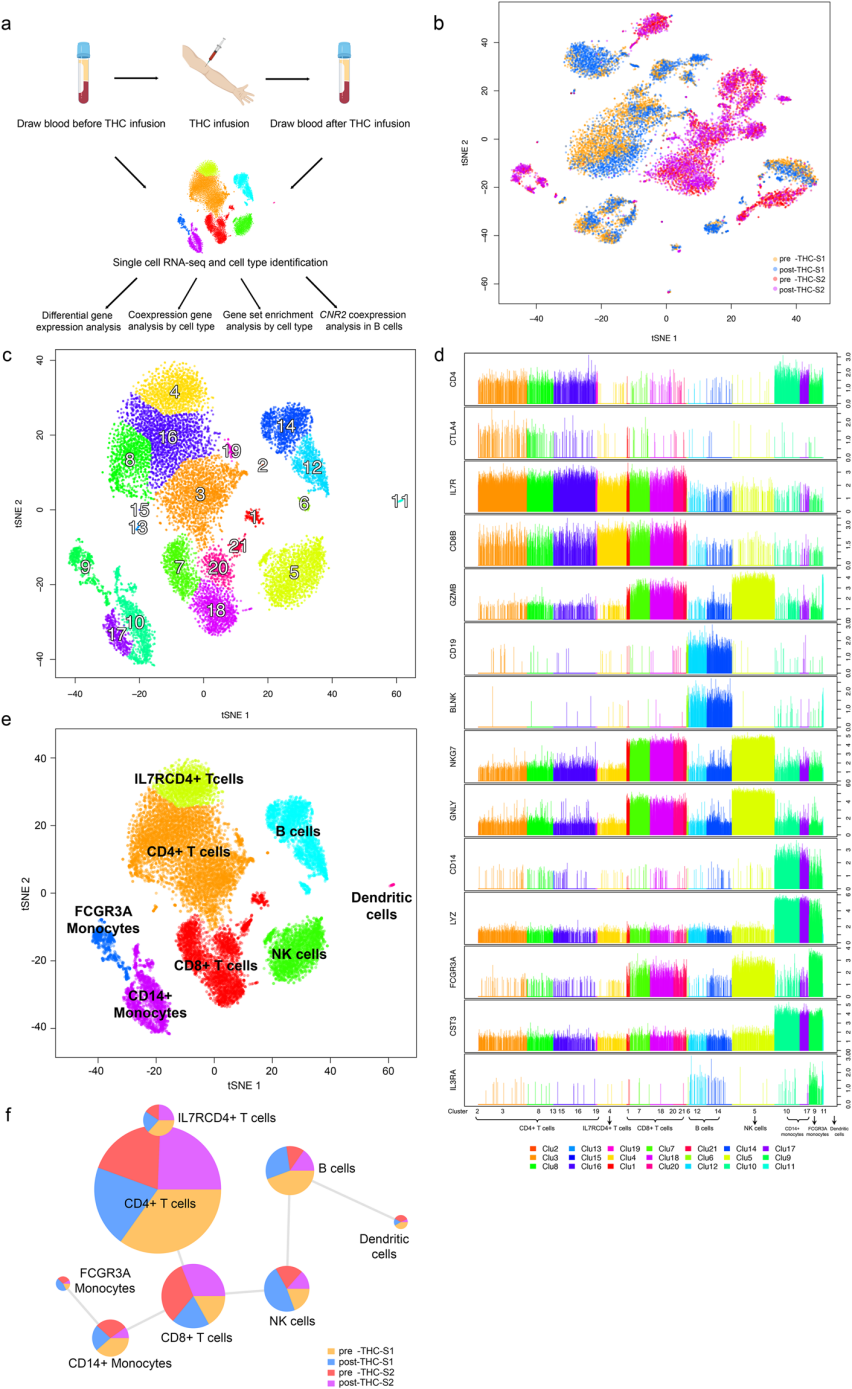
A flow chart illustrating the study design and data analysis strategy. (**a**) Two participants were infused with delta-9-tetrahydrocannabinol (THC). Blood samples were drawn before and 70 minutes after THC infusion. Peripheral blood mononuclear cells (PBMCs) were isolated from each sample and 5,000 cells subjected to single cell RNA-seq using the 10X Genomics platform (figure created with BioRender.com). (**b**) t-SNE plot showing cell transcriptomic clusters of 15,973 PBMCs in four samples pre- and post-THC infusion: pre-THC-S1; post-THC-S1; pre-THC-S2; and post-THC-S2. Cell number in each sample are presented. The plot indicates a batch effect by participants. (**c**) After removal of batch effects by participant, 15,973 cells were clustered into 21 groups by single cell transcriptomic profile. (**d**) Examples of differentially expressed marker genes in each cell cluster. The clusters with similar marker gene profiles in a given cell type were assigned to the same cell type: CD4+ T-cells Clu(2,3,8,13,15,16,19); IL7RCD4+ T-cells Clu(4); CD8+ T-cells Clu (1,7,18,20,21); B cells Clu(6,12,14); NK cells Clu(5) CD14+ monocytes Clu(10,17); FCGR3A monocytes Clu(9) DCs Clu(11). (**e**) Cell type identification by generalized linear modeling (GLM) cell mapping approach and t-SNE plot. A panel of reference genes for each cell type were selected from previously published studies. GLM tested the association of cell type and marker gene expression in each cell. Significance threshold is set at p < 0.01. Each individual cell is assigned to a cell type based on the predominant proportion of marker genes in a given cell type. Small cell clusters are merged into the closest cell type in the t-SNE plot. The cell mapping deconvolutes cells to eight PBMC cell types: CD4+ T-cells, ILR7+/CD4+ T-cells, CD8+ T-cells, B cells, natural killer cells, CD14+ monocytes, FCGR3A monocytes, and DCs. (**f**) Percentage of cell numbers from each sample in a given cell type: pre-THC-S1, post-THC-S1, pre-THC-S2, post-THC-S2.

We profiled the four PBMC samples (two pre-THC and two post-THC) on the 10X Genomics platform^27^. Quality control processing yielded a total of 15,973 cells and 21,430 genes for analyses (Fig. 1a). Before batch effect removal, cells (n = 15,973) were clustered by participant, not by experimental condition (Fig. 1b), indicating that transcriptomic variability between individuals is greater than variability introduced by a single THC dose. We then removed batch effects using Seurat^28^ and surrogate variable analysis^29^ methods and all 15,973 cells clustered into 21 groups (Figs. 1c and S1). To assign cell clusters to cell types, we used a generalized linear model (GLM)-based cell mapping approach with cell-type “marker” genes curated from the literature (see Methods). Briefly, we selected a reference gene panel based on known cell-type-specific gene profiles^27,30^, then used GLM to test the association of gene expression in each cell with the known marker genes (Fig. S2, Table S1). Each cluster was assigned a cell type based on the highest percentage of significant cells (Table S2). Expression of marker genes differed significantly in cell types (Figs. 1d and S2). This approach deconvoluted the 15,973 cells among 21 clusters into eight cell subtypes: CD4+ T-cells (34.6%), IL7RCD4+ T-cells (8.4%), CD8+ T-cells (17.4%), B cells (13.2%), natural killer (NK) cells (12.3%), CD14+ monocytes (10.0%), FCGR3A monocytes (3.9%), and dendritic cells (DC) (0.3%) (Fig. 1e). The proportions of each cell type among the participant samples pre- and post-THC infusion are presented in Fig. 1f and Table S3. This robust cell type identification allowed us to examine THC-regulated gene expression in each cell type.

### Delta-9-tetrahydrocannabinol (THC) alters gene expression shared in multiple cell types of PBMCs

**Figure 2:**
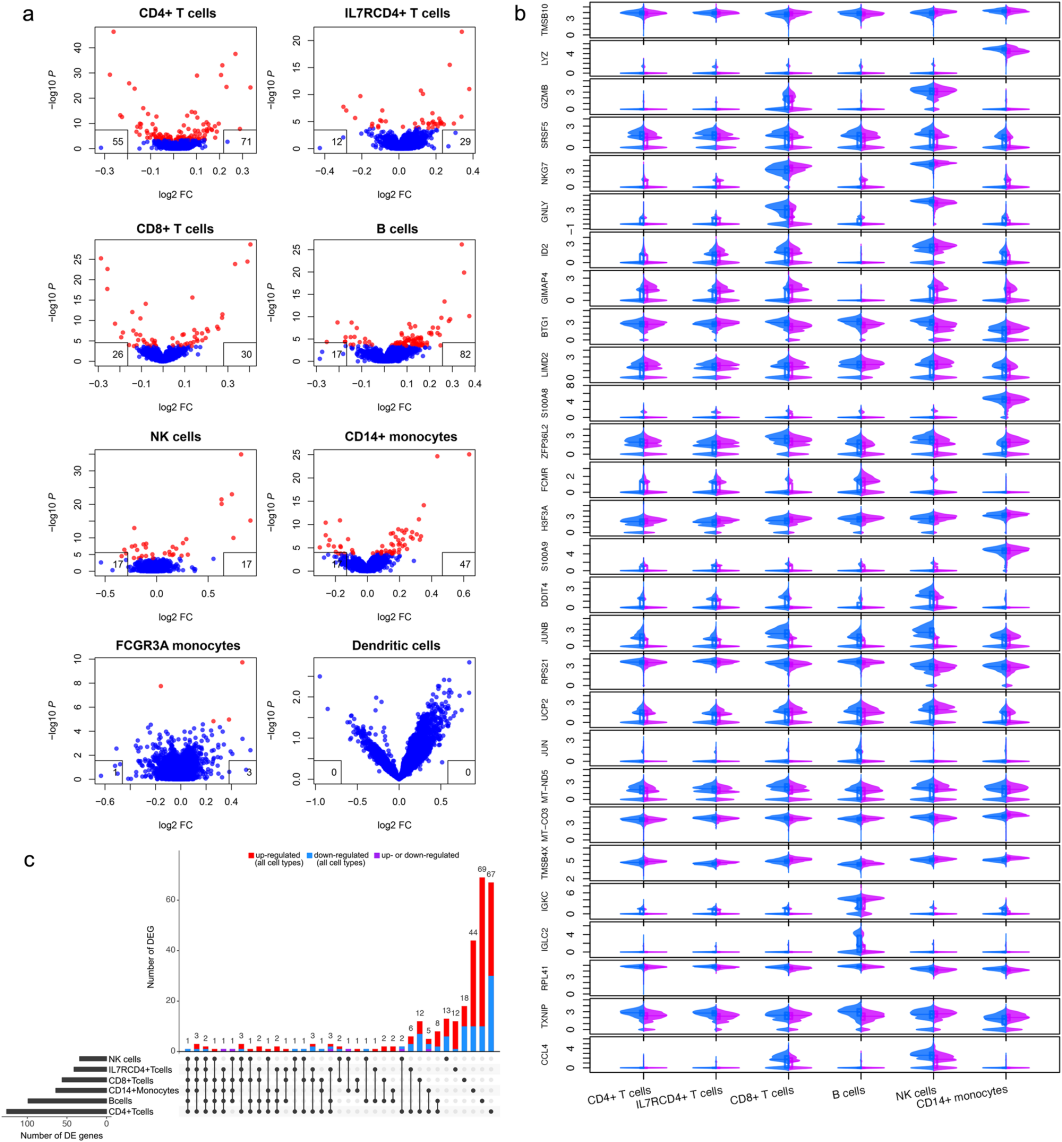
Single cell transcriptome profiling revealed gene expression affected by a single dose of delta-9-tetrahydrocannabinol(THC). (**a**) Linear Mixed Regression analysis identified differential expression of genes affected by THC infusion in eight cell types of peripheral blood mononuclear cells: CD4+ T-cells, ILR7CD4+ T-cells, CD8+ T-cells, B cells, natural killer cells, CD14+ monocytes, FCGR3A monocytes, and DCs. Differentially expressed genes were identified by applying linear mixed model (false discovery rate, FDR < 0.05). Each inset box denotes the number of up-regulated (right box) and down-regulated (left box) genes. No differentially expressed genes(DEGs) were found in DCs. (**b**) Violin plots showing common DEGs between pre-THC and post-THC in at least three cell types. X-axis represents cell type; Y-axis represents expressionlevel for each gene. Blue: pre-THC samples; pink: post-THC samples. (**c**) Number of DEGs in six major cell types. X-axis represents DEGs in each cell type; Y-axis represents number of DEGs. A total of 69 DEGs are shared in at least two cell types, while 225 DEGs are unique for individual cell type (FDR < 0.05). Among shared DEGs in multiple cell types, up(red)- or down(blue) -regulated genes by THC are in the same direction across cell types except three DEGs (purple).

We next applied LMM to detect individual genes affected by THC infusion, with participant included as a random variable to limit confounding effects by individual’s genomic background. We identified 294 transcriptome-wide significant genes in eight cell types affected by THC infusion (false discovery rate, FDR < 0.05) (Fig. 2a). DCs and FCGR3A monocytes were excluded from further analyses because no gene reached transcriptome-wide significance in DCs and both cell types had low cell numbers. Among the 294 DEGs, 69 were observed in at least two cell types while 225 were significant in only one cell type (Fig. 2b; Tables S4–S11). Overall, THC infusion resulted in more upregulation than downregulation in gene expression.

We sought to identify THC-regulated genes common across the six common cell types. We found 28 DEGs in at least three cell types (Fig. 2c). The majority (89%) of the DEGs showed consistent directions of gene expression changes by THC in different cell types; only three genes displayed opposing direction in CD14+ monocytes compared to other cell types (*TMSB4X*, *JUNB, TXNIP*). A group of THC-regulated gene functions are in the domains of immune response and inflammatory process. For example, expression of *S100A9* and *S100A8*, which play prominent roles in the regulation of proinflammatory processes and immune response^31–33^, decreased in response to THC infusion in five cell types. A major HIV-1 suppressive gene, *CCL4*, showed increased expression after THC infusion. THC decreased expression of *GNLY* that is involved in activating antigen presentation^34^. The altered expression of genes involved in humoral immunity were also observed (*IGLC2, IGKC)*. Changes in expression of these genes suggest that THC activated the adaptive immune system shortly after administration, consistent with findings showing an immunomodulatory effect by THC and more complicated than solely immunosuppression^3,8,35–38^.

Among the 28 DEGs shared by at least three cell types, a subset of DEGs supports previous findings that acute THC exposure inhibits cell proliferation and induces apoptosis. Genes responsible for cell death were upregulated (*BTG1*^39^*, DDIT4*^40^*, GZMB*) and genes involving in cell growth and differentiation were down-regulated (*TMSB10*^41^, *RPS21*^42^, *RPL41*^43^ by THC exposure. The alteration of these genes in distinct cell types may indicate potentially deleterious effects of THC on cell differentiation and survival.

We carried out RT quantitative PCR (qPCR) in attempt to validate gene expression differences of common DEGs in at least four cell types using the PBMCs from the same blood samples. Using bulk RNA extracted from the PMBCs, we found that the fold changes of 13 DEGs were highly correlated between bulk PBMCs and CD8+ T-cells (r = 0.76, p = 0.002), and marginally correlated with CD4+ T-cells (r = 0.48, p = 0.09). Figure S3 is a heatmap showing the fold change of gene expression induced by THC by scRNA-seq in six cell types and the fold change of gene expression in bulk RNA by qPCR. This result suggests that THC altering gene expression in 13 common genes is consistent between PBMCs and subtypes of cells. To compare fold changes of DEGs between CD8+ T-cells and PBMCs, we found that expression changes by THC in 9 out of 13 DEGs were concordant. Differential expression of pre- and post-THC in two genes, *TMSB4X and MT-ND5*, were in opposite direction between bulk RNA in PBMCs and scRNA-seq in CD8+ T-cells. Two genes, *ID2* and *ZFP36L2*, were differentially expressed between pre-THC and post-THC by scRNA-seq method, but not by qPCR. Therefore, this experiment validates 70% of DEGs regulated by acute THC administration in PBMCs.

To explore potential mechanisms of THC-regulated gene expression of 294 genes, we performed upstream regulator analysis. We identified more than 2,000 genes that may be involved in regulating 294 gene expression, including genes in cytokine, transcription regulation, signaling pathways (Table S12). Figure S5 presents an example of inferred mechanistic *TNF* gene network involving in THC-regulated gene expression.

### THC perturbs gene expression and KEGG-based gene network in unique cell type and sub-cell clusters

Given that the majority of THC-regulated DEG are unique to each cell type, we then focused on DEGs and gene-gene networks in each cell type. DEGs were determined both in individual clusters in each cell type and among all clusters in each cell type. Here, we conducted a gene network analysis by leveraging gene-gene relationships cataloged in the Kyoto Encyclopedia of Genes and Genomes (KEGG) database^44^. The rationale is to identify gene networks within the biologically known genes or pathways. Thus, the KEGG-based gene network is defined as the inferred gene-gene relationships by KEGG in each cell type. We constructed gene networks independently in pre- and post-THC samples in each cell type (FDR < 0.05). Hub genes in each cell-type based network were defined as a node (gene) with at least four edges (gene links) in at least one condition (pre-, post-THC, or both). We then performed Gene Ontology (GO) term enrichment analysis for each cell-type-specific KEGG-based gene network (FDR < 0.05).

**Figure 3:**
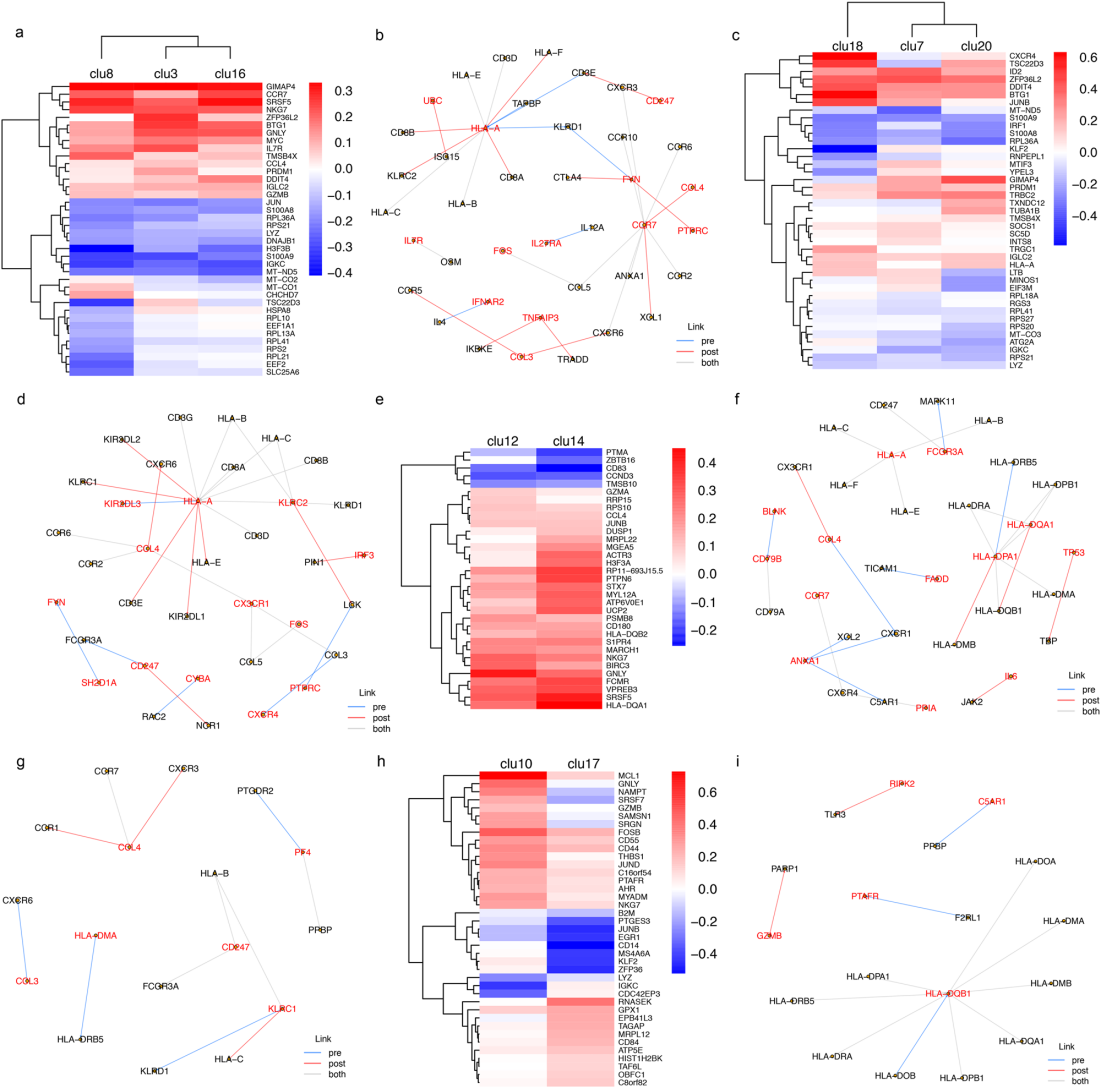
Cell-type cluster based differential gene expression, gene-gene correlation, and biological pathways influenced by delta-9-tetrahydrocannabinol(THC). (**a**) Heatmap showing three subtypes of CD4+ T-cells affected by THC. Color bar represents log2 fold change between post-THC and pre-THC samples. Each row represents significant genes for top 20 genes in each cell cluster. Cluster 8 shows a distinct pattern of differential gene expression from those observed in clusters 3 and 6. (**b**) KEGG-based gene network in CD4+ T-cells. Genes with nominal p < 0.001 from linear mixed regression model are selected to construct a gene network. Gene-gene links were derived from KEGG: Kyoto Encyclopedia of Genes and Genomes – GenomeNet. Significance is set at false discovery rate (FDR) < 0.05. Two hub genes, *CCR7* and *HLA-A*, are differentially expressed genes influenced by THC. (**c**) Heatmap showing three subgroups of CD8+ T-cells affected by THC. (**d**) KEGG-based gene network in CD8+ T-cells. Genes with nominal p < 0.001 in CD8+ T-cells are selected to construct a KEGG-based gene network. In CD8+ T-cells, we observed 35 nodes and 31 edges. One hub gene, *HLA-A*, is differentially expressed by THC. (**e**) Heatmap showing two subtypes of B cells affected by THC. Each row represents significant differentially expressed genes in B cells. Cluster 12 shows a distinct differential gene expression pattern from that of cluster 14. (**f**) KEGG-based gene network in B cells. Genes with nominal p < 0.001 in B cells are selected to construct a gene network based on gene-gene relationship in KEGG. A total of 34 nodes and 28 edges were constructed. Three hub genes, *HLA-A, HLA-DQA1*, and *HLA-DPA1* were identified. (**g**) KEGG-based gene network in natural killer (NK) cells. We observed 17 nodes and 12 edges. One hub gene, *CCL4*, was significantly differentially expressed following THC infusion. (**h**) Heatmap showing two distinct subtypes of CD14+ monocytes (cluster 10 and cluster 17) affected by THC. Color bar represents log2 fold change between pre-THC and post-THC samples. Each row represents significant genes in CD14+ monocytes. (**i**) KEGG-based gene network in CD14+ monocytes. Genes with nominal p < 0.001 in CD4+ T-cells were selected to construct a KEGG-based network. We observed 18 nodes and 13 edges. One hub gene, *HLA-DQB1*, was identified in both pre-THC and post-THC samples.

In CD4+ T-cells, significant DEGs are involved in cytotoxic T-cell activation (*IL7R*^45^), histone modification (*H3F3B*^46^), and transcriptional regulation (*MYC*^47^). We observed three cell sub-groups (cluster 3, 8, and 16; Fig. 3a; Table S13). Cluster 8 showed a distinct DEG pattern from clusters 3 and 16. KEGG-based gene network analysis identified 40 nodes with 33 edges enriched on 14 GO terms including immune response, cell surface receptor signaling pathway, cellular response to stimulus. Two hub genes in the network, *CCR7* (significantly connected with *CCR* [*CCR2, CCR6, CCR10*] and *CXCR* [*CXCR3*, *CXCR4*] family genes) and *HLA-A* (significantly correlated with three other *HLA* genes [*HLA-B, HLA-E, HLA-F*], were significantly affected by THC infusion (Fig. 3b), showing that acute THC exposure perturbed gene-gene relationships in CD4+ T-cells and appears to increase the gene-gene statistical correlation involving cell-mediated immunity.

In CD8+ T-cells, we identified 18 unique DEGs that are involved in immune response and inflammation in response to THC (e.g. *IL32*^48^, *SOCS1*^49^, and *IRF1*^50^). Of note, THC infusion resulted in the differential expression of *CXCR4, TSC22D3, DDIT4, BTG1, JUN* in cluster 18 of CD8+ T-cells (Fig. 3c), suggesting that cells in cluster 18 may function differently in response to THC as compared to the other CD8+ T-cell sub-group. The KEGG-based gene network included 35 nodes that enriched on 12 GO terms (e.g., immune system process), similar to the CD4+ T-cell network. One hub gene, *HLA-A* (strongly correlated with *HLA-B* and *HLA-C* in pre-and post-THC samples), was observed in the network (Fig. 3d; Table S14).

In B cells, genes unique to B cells were observed that are involved in B cell maturation (*VPREB3*^51^), MHC function (*HLA-DQA1*, *HLA-DQA2*), calcium signaling (*CALM2*^52^), Toll-like receptors (*CD180*^53^), and response to environmental stress via activating MAP kinase MAPK1/ERK2 (*DUSP1*^54^). Consistent with *in vitro* findings, we found that acute THC exposure reduced expression of *BCL2* in B cells^45^. The majority of DEG originated from cluster 14 (Fig. 3e; Table S15), with only 8 DEGs from cluster 12, which were all upregulated by THC. In the KEGG-based gene network, we found 34 nodes with 28 edges (Fig. 3f) enriched on GO pathways including MHC protein complex, immune response, peptide antigen binding. Four hub genes were identified that differed in response to THC (*HLA-A and HLA-BQA1) HLA-DPA1* [became more strongly correlated with *HLA-DQB1* post-THC]*, ANX1* [connected to *CXCL1*, *CXCR4*, *XOL2* in pre-but not post-THC samples]). Notably, two B cell marker genes (*BLNK*, *CD79B*) were correlated in post- but not pre-THC samples.

In NK cells, which is defined by one cluster, many DEGs (e.g. *DDIT4, CCL4, BTG1 ID2*) are involved in immune response and cell proliferation. One DEG unique to NK cells, *CD53*, was downregulated by THC. Genes in the NK cell network (17 nodes and 12 edges; Fig. 3g) were enriched on 17 pathways including chemokine-mediated signaling pathway, inflammatory response, and chemokine receptor activity. Notably, *KLRC1* regulates specific humoral and cell-mediated immunity and is implicated in the recognition of the MHC class I HLA molecules in NK cells and was correlated with *HLA-C*, which increased in post-THC samples. No hub genes were observed.

In CD14+ monocytes, which were composed of two subgroups (cluster 10 and 17; Fig. 3h; Table S16), THC infusion resulted in unique 44 DEGs, including genes regulating cell fate (e.g. *MCL1*, *FOSB*, *MYADM*). Genes in the CD14+ monocyte KEGG-based gene network (18 nodes and 13 edges; Fig. 3i) were enriched on pathways including MHC class II protein complex, antigen processing, immune response, and cellular response to interferon-gamma. One hub gene, *HLA-DQB1*, was observed.

These observations from individual gene and KEGG-based gene networks in different cell types suggest a diverse functional response to acute THC exposure in heterogenous immune cells.

Significant pathways for all KEGG-based gene networks in each major cell type are presented in Tables S17–S22.

### Common biological pathways including immune response and cell survival/apoptosis are associated with THC administration

**Figure 4:**
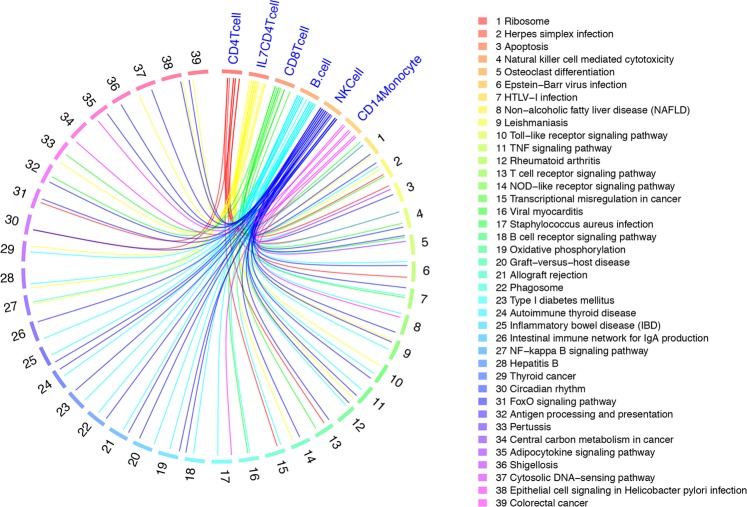
Circus plot showing gene set enrichment analysis using KEGG annotations reveal 39 biological pathways shared in at least 2 cell types. KEGG: Kyoto Encyclopedia of Genes and Genomes – GenomeNet. Significant pathway is declared at false discovery rate (FDR) < 0.05. The legend is the name of each pathway corresponding to each spoke of the circular plot.

We subsequently performed a cell-type based gene set enrichment analysis (GSEA). We found 39 significant KEGG pathways in at least two cell types (Fig. 4; Tables S24–S31); significant pathways were involved in the domains of immune response, inflammation, and cell survival and apoptosis. Several pathways associated with autoimmune disease (e.g., rheumatoid arthritis) were significant in multiple cell types. The ribosomal pathway, which plays a role cell growth and cellular response to stress, was the most significantly enriched pathway across all five cell types. These results further support the effects of THC on functional domains in immune regulation that have also been associated with immunological disease, although the causality of these relationships is unknown.

### THC changes *CNR2* co-expression network in B cells

**Figure 5:**
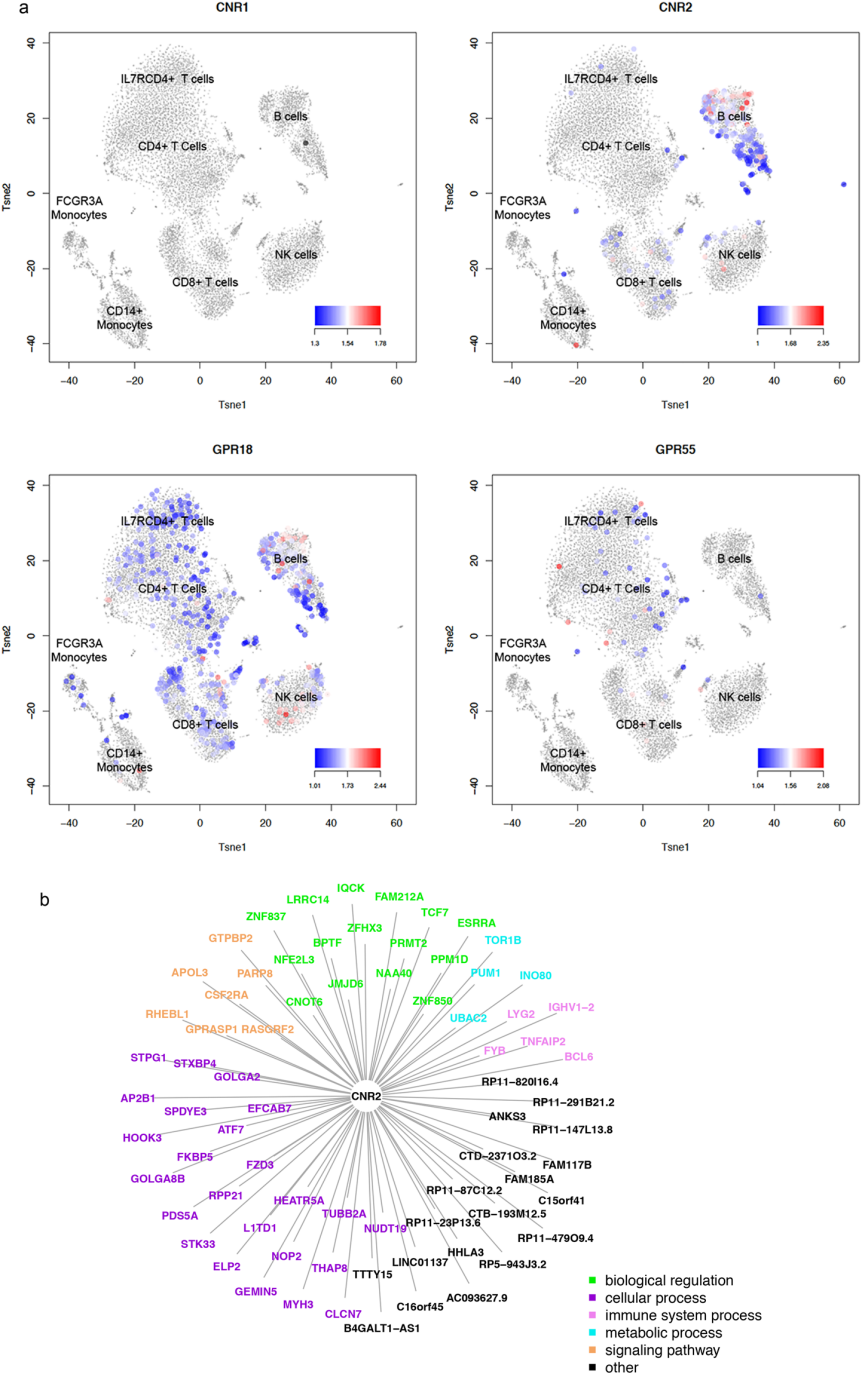
THC receptor gene expression and co-expression network in B cells. (**a**) t-SNE plot showing THC receptor gene expression in distinct cell types of peripheral blood mononuclear cells. *Small amount of CNR1* expression is observed in any of the cell types. *CNR2* is highly expressed in B cells, followed by nature killer cells, CD8+ T-cells, and minimally expressed in CD4+ T-cells. *GPR55* is highly expressed in CD4+ T-cells more than other cell types. *GPR18* is widely expressed in peripheral blood mononuclear cells. (**b**) *CNR2* expression is significantly correlated with 74 genes in B cells. The genes co-expressed with *CNR2* are involved in functions of biological regulation, cellular process, immune system process, metabolic process, and signal functions.

Finally, we were interested in understanding how cannabinoid receptor genes co-expressed with other genes in each cell type following THC administration. As expected, *CNR2*, encoding for CBR2, was highly expressed in B cells, followed by NK cells, then CD8+ T-cells, and lowest in CD4+ T-cells (Fig. 5a)^55^. Little *CNR1* expression was detected in any of the cell types. *GPR55* (cannabinoid receptor 3^56^) showed the highest expression in CD4+ T-cells. We observed gene co-expression between *CNR2* and 84 and 74 genes in pre- and post-THC samples, respectively (Tables S31–S32). The co-expressed genes in post-THC B cells were enriched on functional domains of immune processes, biological regulation, cell proliferation, signaling, and response to stimulus (Fig. 5b). Interestingly, little overlapped genes in two *CNR2* co-expressed network, suggesting that THC significantly reorganizes *CNR2* connected gene-gene relationships in B cells (test of overlapping genes between two group p = 0.302).

## Discussion

Our results from a well-controlled human study demonstrate transcriptomic regulation in distinct immune cells following administration of a single dose of THC. In our model, THC increased subjective effects (“high”, “stoned”) and heart rate consistent with the known effects of cannabis. Thus, THC altered gene expression at a dose that produces effects consistent with the known effects of cannabis. Our study design and analytical approach enabled the identification of common and cell-type-specific DEGs regulated by THC. The cell-type-specific gene and co-expression patterns revealed by scRNA-seq showed little overlap across cell types, which would have been obscured by bulk RNA-seq analysis. Cell type-based DEGs, co-expression networks, and GSEA revealed important THC effects on immune function, cytokine production, signal transduction, and cell apoptosis and survival.

We used within-subject design and mixed regression model to minimize confounding factors on THC’s effects and to control high individual transcriptome variability. The approach also reduces potential batch effects across different individuals and experimental batches. Subjective, behavioral and cognitive tests confirm that the dose of THC was relevant to but limited the confounding effects of other compounds in cannabis. These strengths enabled us to reveal differentially expressed genes from highly variable transcriptomes. In addition, we applied GLM method for cell-type identification combining with knowledge-based cell markers. The approach identified biologically meaningful cell-types sub-clusters in major cell types in PBMCs. The sub-clusters present specific DEG patterns altered by THC and different co-expression networks regulated by THC, suggesting that the sub-clusters may be functionally distinct cell types. The biological verification of sub-clusters is warranted in a future study.

Although the majority DEGs were cell-type-specific, we observed top significant differentially expressed genes shared across multiple cell types. Importantly, we validated the top 18 common genes affected by THC in at least four cell types in bulk RNA from PBMCs by RT q-PCR in the same samples. The functions of common DEGs were relevant to the biological pathways shared in multiple cell types as well. These common DEGs present almost identical direction by THC regulation, suggesting that THC’s immune response is involved in important genes to maintain immune response across different cell types. We identified more up-regulated genes than down-regulated genes by THC, which is expected after acute THC administration. Importantly, the large number of DEGs shows cell-type-specific fashion and a single low dose of THC perturbates cell-type-specific co-expression network. The complexity of THC’s cell-type-specific effect raises a challenge of therapeutic treatment using cannabis for medical diseases.

### Limitations

The results of this exploratory, pilot study should be considered in the context of its limitations and strengths. First, the findings need to be validated in a larger sample. Second, while the IV THC paradigm offers excellent experimental control, it has limited ecologically validity. Therefore, the effects of acute administration of smoked or vaporized cannabis should be studied. Third, while the effects of a single dose of THC were studied, given the known biphasic effects of cannabinoids, the dose-related effects of cannabinoids on gene expression need to be investigated. Furthermore, while we demonstrated the changes in gene expression at one time point after the administration of THC, the full temporal profile of changes in gene expression need to be studied. Fourth, while the effects of a single dose of THC were demonstrated, the effects of chronic exposure to medicinal and recreational cannabinoid need to be studied. Fifth, while the effects of THC in healthy subjects was demonstrated, how cannabinoids people with altered immune function (e.g., HIV) or chronic inflammatory conditions (e.g., rheumatoid arthritis) needs to be studied. Sixth, the next logical step is to investigate the precise mechanisms underlying THC altered gene expression observed in the exploratory study. For example, CHIP-qPCR analysis to detect histone markers may further enhance our understanding of the mechanism of the identified immune/inflammation-related genes function^57^. Finally, while the effects of THC on transcriptomic profiling and differential gene expression has been demonstrated, the effects of the other constituents of cannabis needs to be studied.

Nevertheless, the study provides a foundation for future studies of cell-type-specific immunologic effects of cannabis and cannabinoid constituents. These findings highlight the complexity of cannabinoid effects on immune function and nuance of immune cell types that may have medical relevance.

## Methods

The study was conducted at the Neurobiological Studies Unit (VA Connecticut Healthcare System, West Haven, CT) with the approval of the Institutional Review Boards at VA Connecticut and Yale University, and the Protocol Review Committee of the Department of Psychiatry, Yale University. The study was amended to include prospective measures addressing safety. All methods were performed according to the relevant guidances and regulations by Yale University and VA Connecticut Healthcare System.

### Participants

Two healthy participants were recruited from the community by advertisements. Both participants were male, 21-year old, and of European American descent. Subjects were informed about the potential for psychosis, anxiety, and panic. After obtaining informed consent, subjects underwent a structured psychiatric interview for DSM-IIIR^58^ and were carefully screened for any DSM-IV Axis I or Axis II lifetime psychiatric or substance abuse disorder (excluding nicotine) and family history of major Axis I disorder. The history provided by subjects was confirmed by a telephone interview conducted with an individual (spouse or family member) identified by the subject prior to screening. In order to avoid exposing cannabis-naïve individuals to a potentially addictive substance, only subjects who had been exposed to cannabis but did not meet lifetime criteria for a cannabis use disorder were included. Past month cannabis use was quantified using a time-line-follow-back approach. Finally, subjects underwent a general physical and neurologic examination, EKG, and laboratory tests (serum electrolytes, liver function tests, complete blood count with differential and urine toxicology). Subjects were instructed to refrain from caffeinated beverages, alcohol, and illicit drugs from 2 weeks prior to testing until study completion. Urine toxicology was conducted on the morning of each test day to rule out recent illicit drug use.

### Procedure

Subjects received 0.03 mg/kg of THC, the principal active ingredient of cannabis^23,24^. This dose equivalent to 2.1 mg in a 70 kg individual has been shown in previous studies to produce effects consistent with the known effects of cannabis, in a safe manner^59–61^. THC was administered on its own, without the >450 other chemical constituents of cannabis because THC is the principal active constituent of cannabis and the other chemical constituents could render the results challenging to interpret. The intravenous route of administration was chosen to reduce inter and intraindividual variability in plasma THC levels with the inhaled route^62^. Timeline of behavioral assessment and blood draw is presented in Fig. S5. Subjects were attended to by a research psychiatrist, a research nurse, and a research coordinator. Clear ‘stopping rules’ were determined a priori and rescue medication (lorazepam) was available if necessary. Medical condition and psychiatric status of participants were monitored closely during and after THC challenging. Subjective and clinical ratings were repeatedly assessed.

### Medical and behavioral assessment

Vital signs, Cannabis intoxication, Psychotic symptoms, Perceptual alteration, and Cognitive test battery were measured prior, during, and post THC infusion as illustrated in Fig. S5.

Physiological and psychoactive assessments of two participants are presented in Table S33.

### Single cell RNA sequencing in 10X genomics platform

PBMCs from pre-THC (N = 2) and post-THC samples (N = 2) from fresh whole blood were isolated at the same time using a standard protocol. The cells were washed twice with phosphate-buffered saline containing 0.04% bovine serum albumin, and the final cell concentration was adjusted to 1000 cells/mL for library preparation. 5000 cells/sample were prepared for single cell capture.

#### Sequencing data processing and quality control

We used the 10X Genomics Chromium Single Cell 3′ v2.0 platform to prepare individually barcoded single cell RNA-Seq libraries following the manufacturer’s protocol. Library size was confirmed with Agilent Bioanalyzer High Sensitivity DNA assay (PN: 5067–4626), Invitrogen dsDNA HS qubit assay to evaluate dsDNA quantity (PN: Q32854), and KAPA qPCR analysis (KAPA Biosystems LIB Quant Kit, Illumina/LC480, PN: KK4854) to evaluate the quantity of sequencable transcripts.

#### Flow cell preparation and sequencing

Sample concentrations are normalized to 10 nM and loaded onto Illumina Rapid flow cell at a concentration that yields 150 M passing filter clusters per lane. Samples are sequenced using paired-end sequencing on an Illumina HiSeq. 2500 according to Illumina protocols. The 8 bp index is read during an additional sequencing read that automatically follows the completion of read 1. Data generated during sequencing runs are simultaneously transferred to the YCGA high-performance computing cluster. A positive control (prepared bacteriophage Phi X library) provided by Illumina is spiked into every lane at a concentration of 0.3% to monitor sequencing quality in real time.

#### Data analysis and storage

Signal intensities are converted to individual base calls during a run using the system’s Real Time Analysis (RTA) software. Base calls are transferred from the machine’s dedicated personal computer to the Yale High Performance Computing cluster via a 1 Gigabit network mount for downstream analysis. Primary analysis - sample de-multiplexing and alignment to the human genome - is performed using Illumina’s CASAVA 1.8.2 software suite. The Cell Ranger Single-Cell Software Suite (versions 2.0.0 and 2.1.0 for the discovery and validation patients respectively) were used to perform sample demultiplexing, barcode processing and single-cell gene counting (http://10xgenomics.com/). The gene-cell matrix was generated for the following analysis.

#### Data normalization

Only genes with at least one UMI count detected in at least one cell were retained for analysis Single cells were excluded when >10% of reads mapped to mitochondrial RNA to ensure that all of the single cells originated from nucleated cells. The cells with fewer than 370 expressed genes or possible doublet cells (>100,000 reads) were also discarded. Applying these three criteria resulted in retention of 21,430 genes and 15,973 single cells for downstream analysis.

Data normalization was performed using a standard protocol in Seurat^28^. An exploratory analysis showed cells clustered by each participant. Batch effects were then removed using an empirical Bayesian framework (ComBat function in R package sva) with the individual labeled as the random variable. The normalized data was dimensionally reduced in two dimensions using t-distributed stochastic neighbor embedding (t-SNE) with a perplexity parameter of 20 and 3000 iterations after an initial principle component analysis. The distance matrix of the single cells was computed with the output of t-SNE and converted into a graph. Cluster_louvain function was used by default options (https://igraph.org/r/doc/cluster_louvain.html). Then, the graph was clustered with the cluster_louvain function in R package *igraph*, which implements the multi-level optimization of modularity algorithm for finding community structure.

#### Cell type identification

A gene set of the cell type markers in PBMC was manually curated from the literature. A binary cell type matrix with cell types as columns and genes as rows was generated with value 1 representing a marker in a cell type and value 0 denoting non-markers for a cell type. The generalized linear model (GLM) was constructed by deciding on one vector, representing one cell type as a response, of the binary matrix and corresponding gene expressions as explanatory variables in one cell. In details, ${\boldsymbol{Y}}={({y}_{gc})}_{G\times C}$ is the binary cell type matrix of *G* marker genes of *C* cell types and ${\boldsymbol{X}}={({x}_{gs})}_{G\times S}$ is the gene expression matrix of *G* marker genes of *S* single cells. For the *g*^*th*^ gene, ${y}_{gc}=0$ or 1 denotes the cell type-specific gene indicator of *c*^*th*^ cell type and *x*_*gs*_ denotes the gene expression of *s*^*th*^ single cell, where $g=1,\ldots ,G$, $c=1,\ldots ,C$, and $s=1,\ldots ,S$. The linear probability model (LPM) was conducted for our study,$\,{{\boldsymbol{y}}}_{{\boldsymbol{c}}}={\beta }_{0cs}+{\beta }_{1cs}{{\boldsymbol{x}}}_{{\boldsymbol{s}}}+{{\boldsymbol{\varepsilon }}}_{{\boldsymbol{cs}}}$, where ${{\boldsymbol{y}}}_{{\boldsymbol{c}}}={({y}_{1c},\ldots ,{y}_{Gc})}^{T}$ and ${{\boldsymbol{x}}}_{{\boldsymbol{s}}}={({x}_{1s},\ldots ,{x}_{Gs})}^{T}$ are the columns of matrix ${\boldsymbol{Y}}$ and ${\boldsymbol{X}}$, represent binary cell type vector and single cell gene expression vector, respectively. ${{\boldsymbol{\varepsilon }}}_{{\boldsymbol{cs}}}={({\varepsilon }_{1cs},\ldots ,{\varepsilon }_{Gcs})}^{T}$ is a random error vector, where ${\varepsilon }_{gcs} \sim N(0,\,{\sigma }^{2})$. The hypothesis of interest to be tested is ${H}_{0cs}:\,{\beta }_{1cs}=0\,$vs ${H}_{1cs}:\,{\beta }_{1cs}\ne 0$. One statistical value (t value) and p value is obtained for each cell and cell type; *t*_*cs*_ and *p*_*cs*_ denote the test statistic value and P-value, respectively for the *c*^*th*^ cell type of *s*^*th*^ single cell.

We used the cutoff, p < *p*_0_ and t value >0 to assign one cell to one cell type, where *p*_0_ is a predefined cut-off value (*p*_0_ = 0.01 in this study). Then, the proportion of each assigned cell type was calculated for every cluster. The dominant proportions are used to assign one cluster to one cell type.

$${P}_{c}^{k}=\frac{{\sum }_{s\in {S}_{k}}(I({t}_{cs} > 0)\cdot I({p}_{cs} < {p}_{0}))}{{N}_{k}},$$

where ${P}_{c}^{k}$ denotes the proportion of the *c*^*th*^ cell type in the *k*^*th*^ cluster. *S*_*k*_ is the set of cell indices for clustering *k*, with ${N}_{k}=|{S}_{k}|$ being the total number of cells in the *k*^*th*^ cluster. Finally, the cell type for each cluster was confirmed manually by cell type marker gene expressions mapping on the 2D t-SNE plot. The cell clusters mapping to the same cell type were merged for downstream analysis.

### Statistics

All statistical analyses were performed using R version 3.5.1 (R Foundation, https://www.r-project.org) and RStudio version 1.1.453 (https://www.rstudio.com).

#### Differential gene expression

We applied a linear mixed regression model to identify genes associated with THC infusion. To control transcriptomic variation between two participants, we used participant as a random effect. Transcriptome-wide significance was set at false discovery rate (FDR) < 0.05.

#### KEGG-based gene network analysis

Here, gene network was defined as significant gene-gene correlation in each given cell type leveraging the relationship among genes in KEGG database. Using this supervised gene network analysis approach, we tested whether gene links in KEGG were significant in each cell type using linear regression model. Significant KEGG-based network was set at FDR < 0.05. The analysis was performed separately in pre-THC and post-THC samples to test if THC alters gene-gene correlation.

#### Gene Ontology (GO) enrichment analysis

Genes in each co-expression network in major cell types were tested the enrichment on GO terms using The DAVID Gene Functional Classification Tool^63^. Significant level was set at FDR < 0.05.

#### Cell type-based gene set enrichment analysis

We separately analyzed gene set enrichment in each cell type using KEGG database. Differentially expressed genes in a given cell type with nominal p < 0.001 were selected. Significant pathway was set at FDR < 0.05.

Co-expression of cannabinoid receptor genes with other genes Four cannabinoid receptor genes, *CNR1, CNR2, CPR18*, and *GPR55* were tested differential expression across seven major cell types using linear mixed regression model. Correlation of *CNR2* with other genes in B cells was performed in pre- and post-THC samples independently. Significant correlation was set at FDR < 0.05. We tested for statistical significance of the overlap between the two groups (pre- and post-THC) of genes (http://nemates.org/MA/progs/overlap_stats.cgi).

## Supplementary information

Supplementary figures.

Supplementary tables.

## Data Availability

Single cell transcriptome data have been deposited in Gene Expression Omnibus and are available under project number GSE130228.
