# Supplementary material for: Single-cell Transcriptome Mapping Identifies Common and Cell-type Specific Genes Affected by Acute Delta9-tetrahydrocannabinol in Humans
Source: Sci Rep. 2020 Feb 26;10:3450. doi: 10.1038/s41598-020-59827-1 (PMC7044203; doi:10.1038/s41598-020-59827-1)
Supplement: Supplementary file 1 — Supplementary figures. [file 41598_2020_59827_MOESM1_ESM.docx]

**Supplementary**

**Single-cell Transcriptome Mapping Identifies Common and Cell-type Specific Genes Affected by** **Acute Delta9-tetrahydrocannabinol in Humans**

Ying Hu^1^, Mohini Ranganathan ^2,3^, Chang Shu ^2,3^, Xiaoyu Liang ^2,3^, Suhas Ganesh ^2,3^, Awo Osafo-Addo ^2,3^, Chunhua Yan ^1^, Xinyu Zhang ^2,3^, Bradley E Aouizerat ^4,5^, John H Krystal ^2,3^, Deepak C. D’Souza^2,3^, Ke Xu ^2,3*^

1. Center for Biomedical Information and Information Technology, National Cancer Institute, MD, U.S.A.

2. Department of Psychiatry, Yale School of Medicine, New Haven, CT, 06516

3. Connecticut Veteran Healthcare System, West Haven, CT, 06515

4. Bluestone Center for Clinical Research, College of Dentistry, New York University, NY, 10010

5. Department of Oral and Maxillofacial Surgery, College of Dentistry, New York University, NY, 10010

Please direct all correspondence to:

* Ke Xu, MD, PhD

Associate Professor of Psychiatry

Yale School of Medicine

Email: ke.xu@yale.edu

Telephone: 203-932-5711x7430

**Supplementary Figures**

**Figure S1. tSNE plot showing cell clusters after removed batch effects**

**
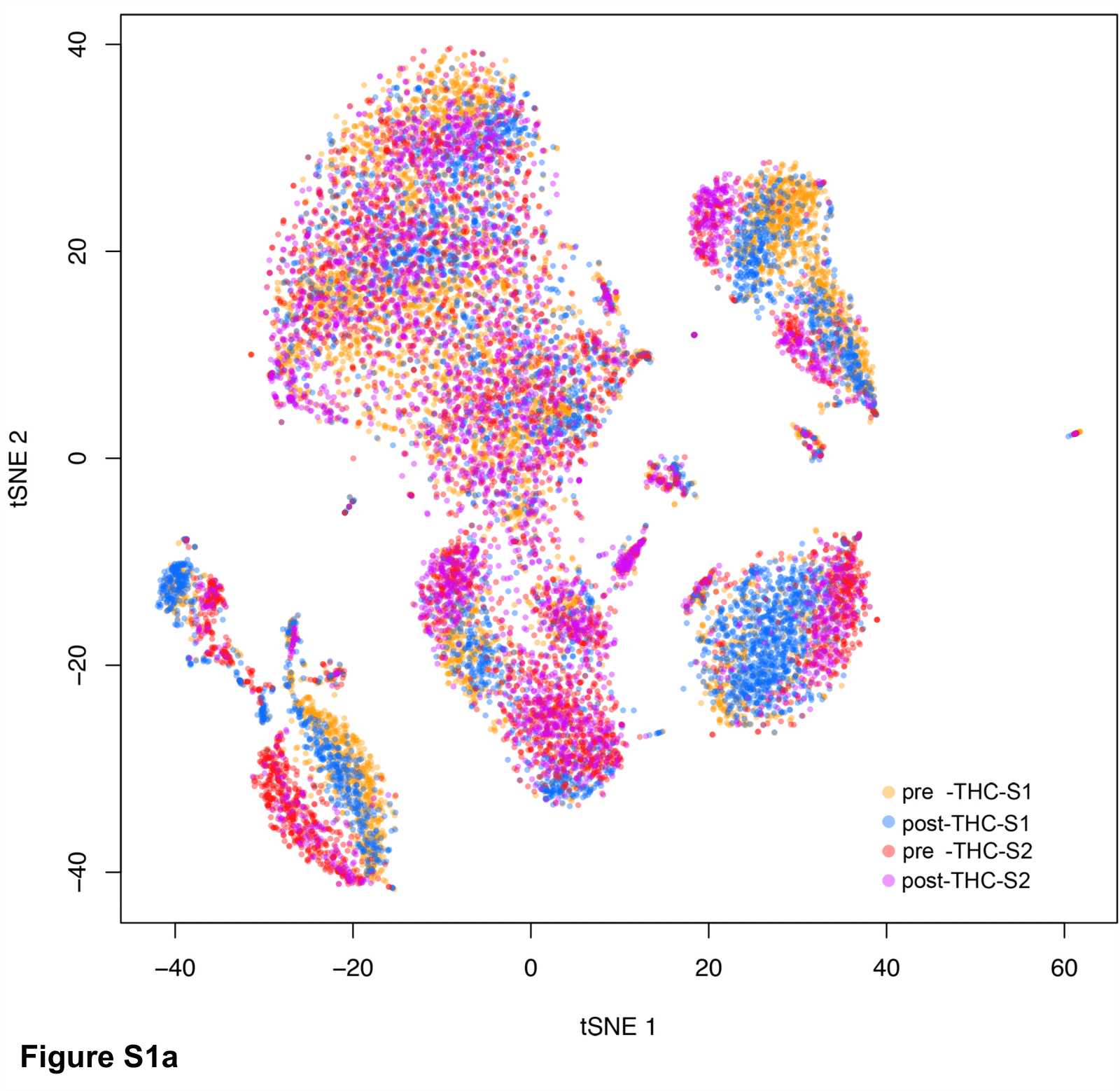
**

**Figure S1a**. tSNE plot for four samples of pre- and post-THC infusion.


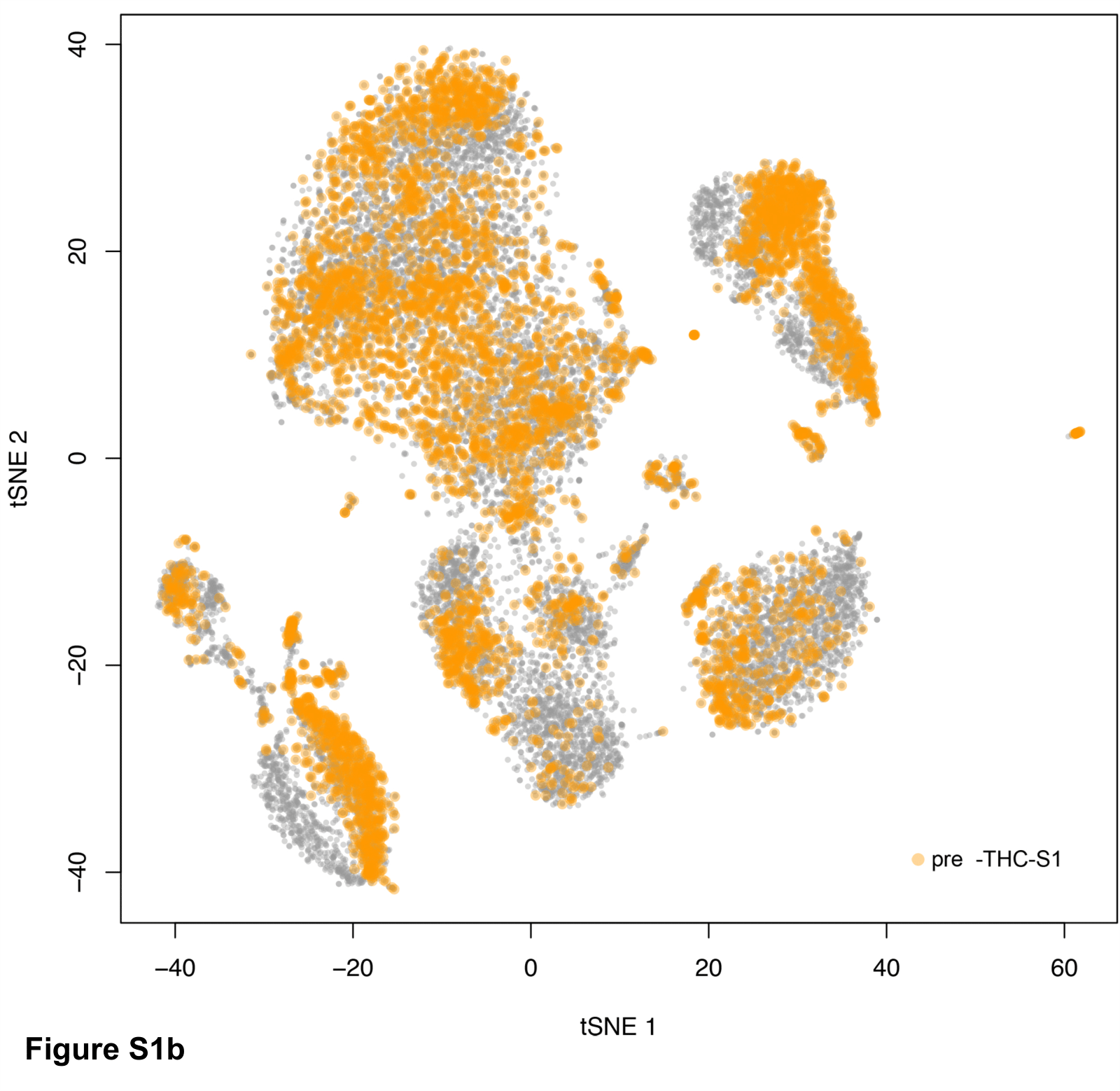


**Figure S1b**. tSNE plot for pre-THC infusion of subject 1


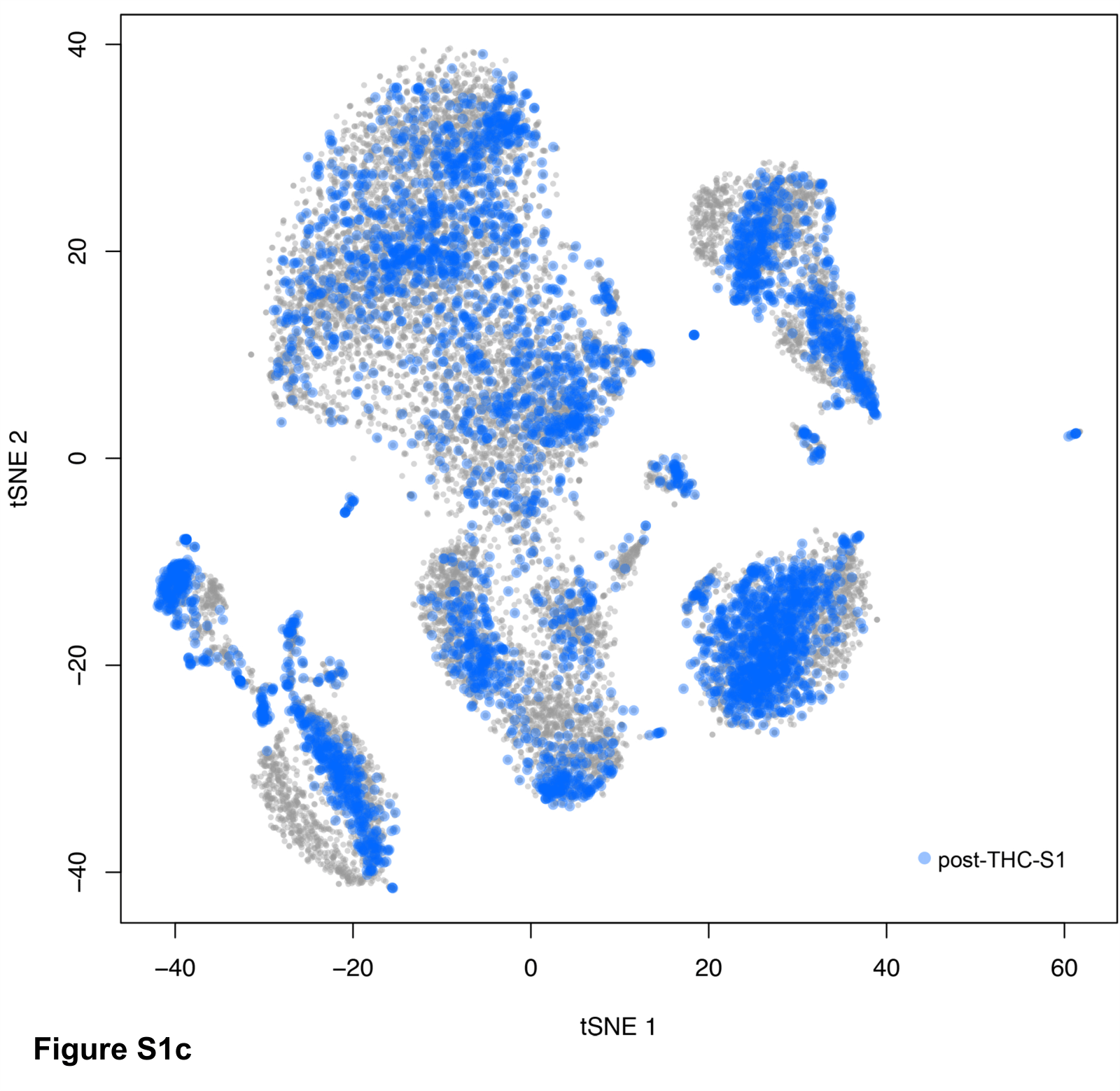


**Figure S1c**. tSNE plot for post-THC infusion of subject 1


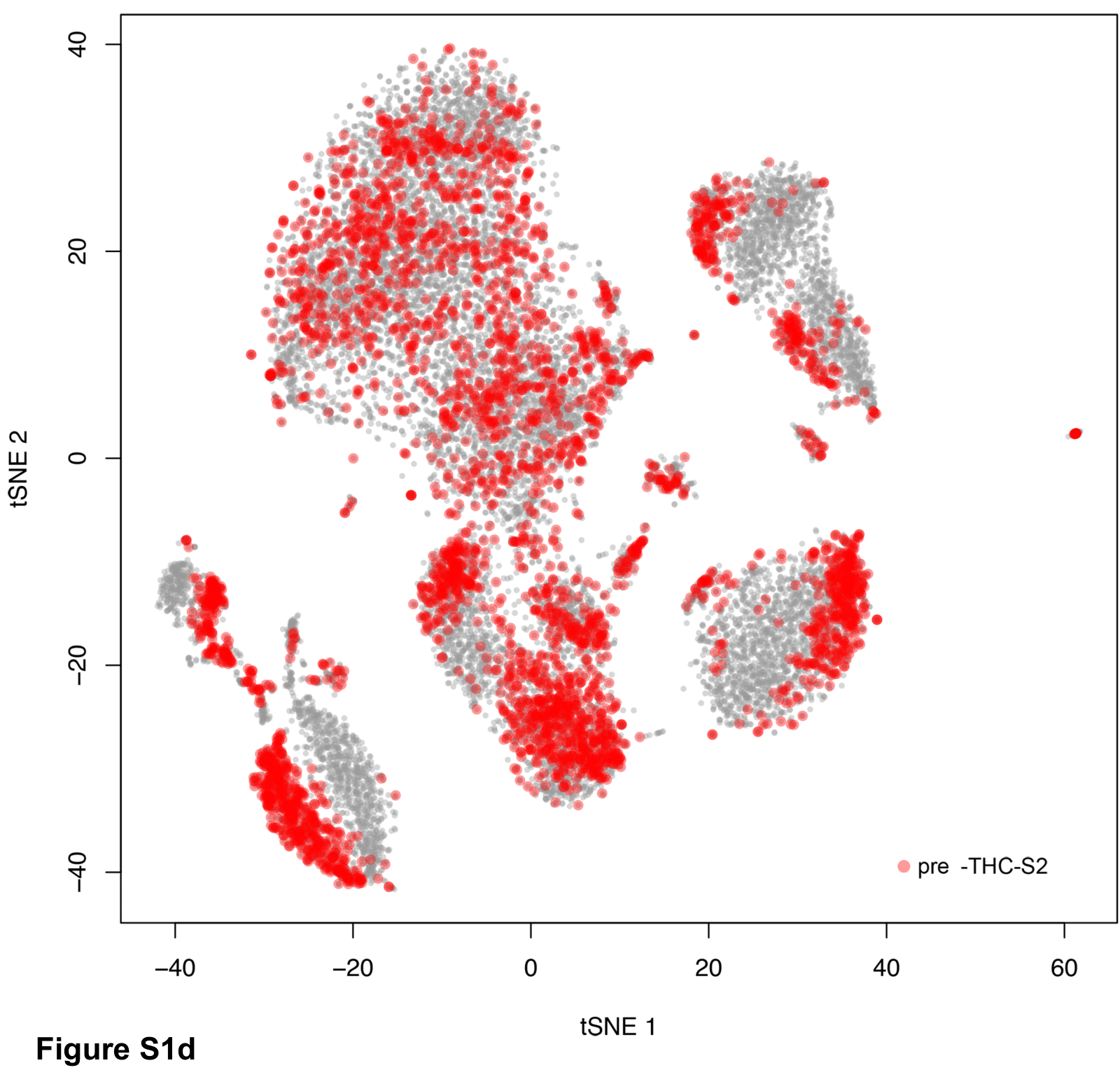


**Figure S1d**. tSNE plot for pre-THC infusion of subject 2


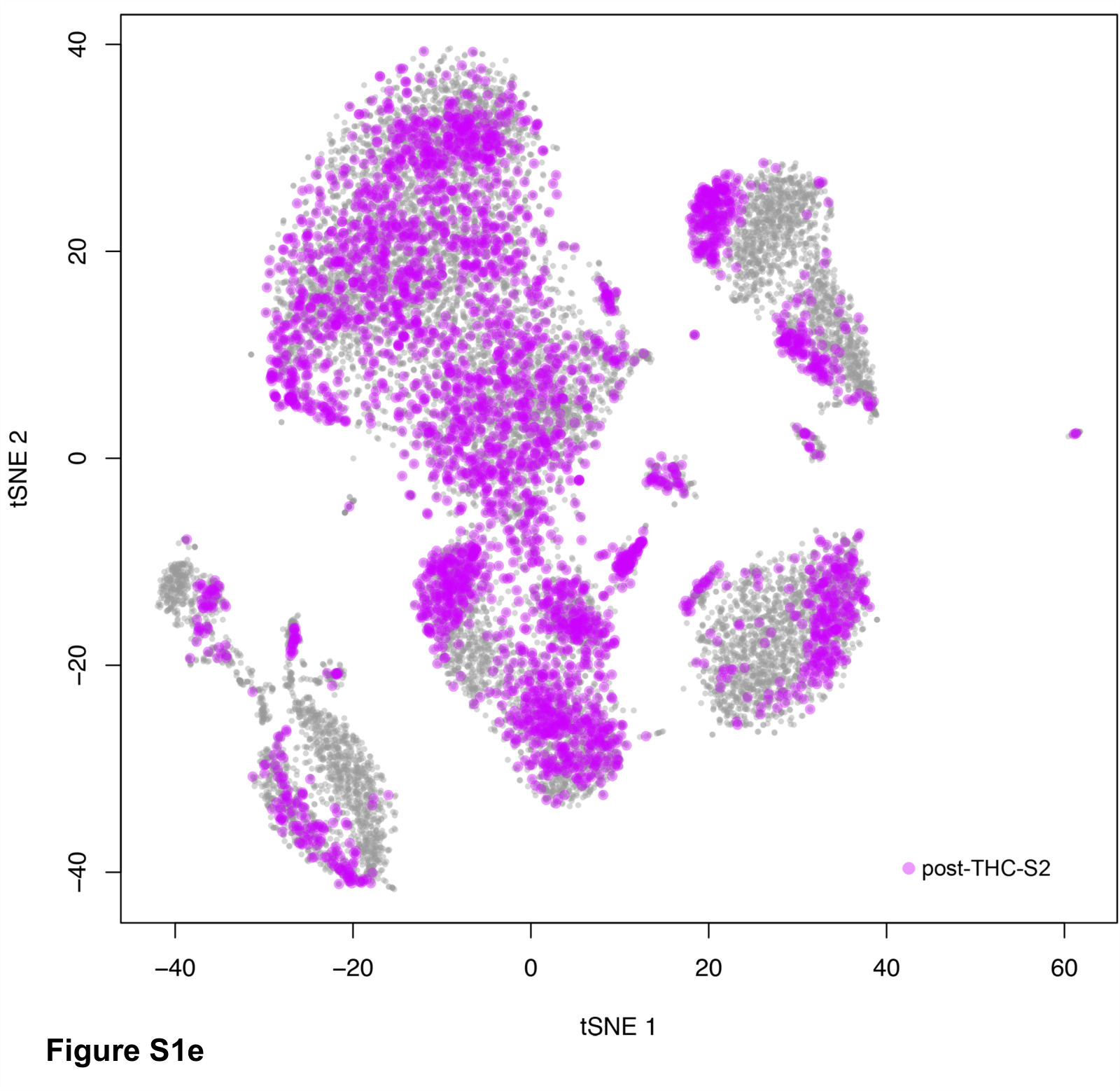


**Figure S1e**. tSNE for post-THC infusion of subject 2.

**Figure S2. Sample marker gene expression in each cell type of 15,973 peripheral blood mononuclear cells**

**
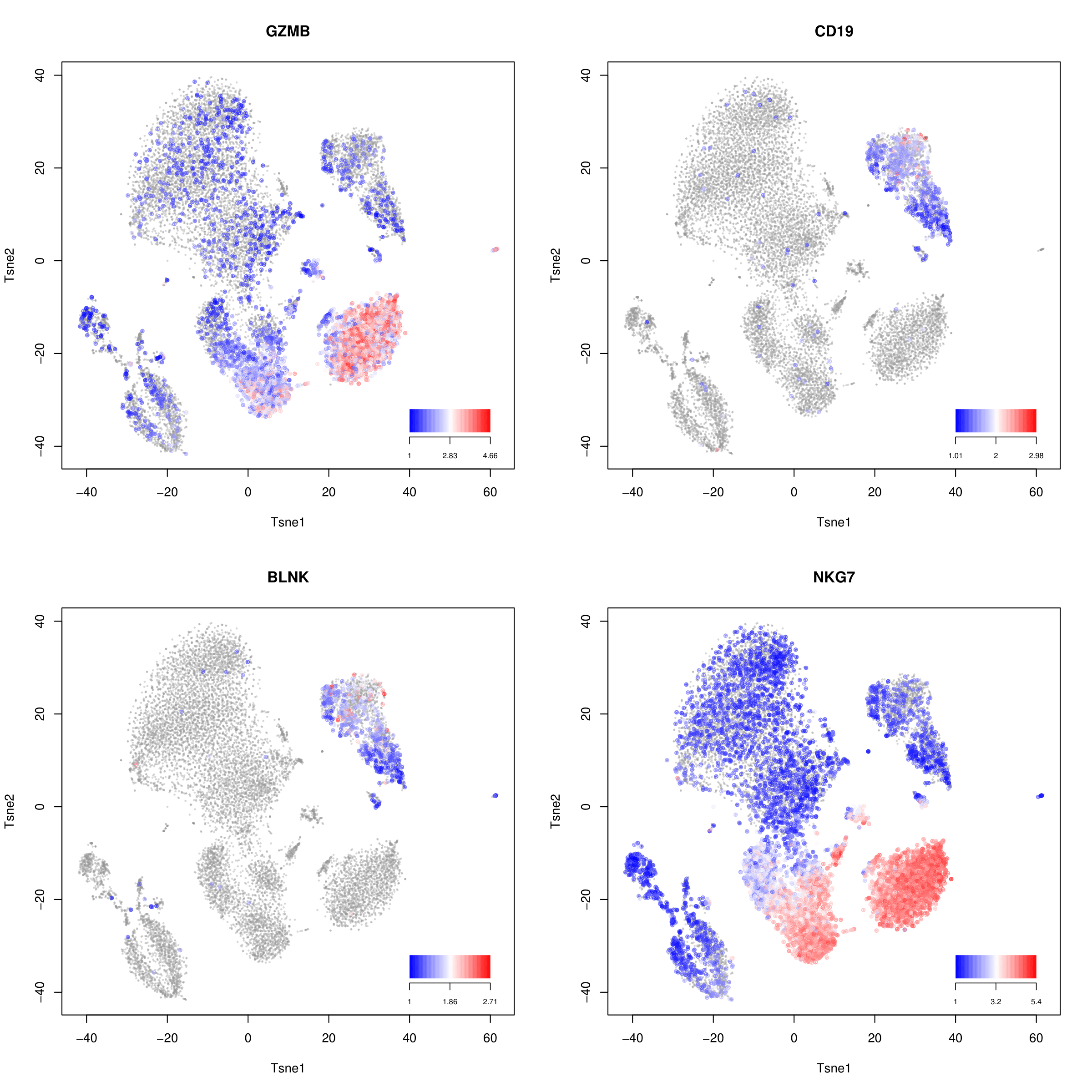
**

**
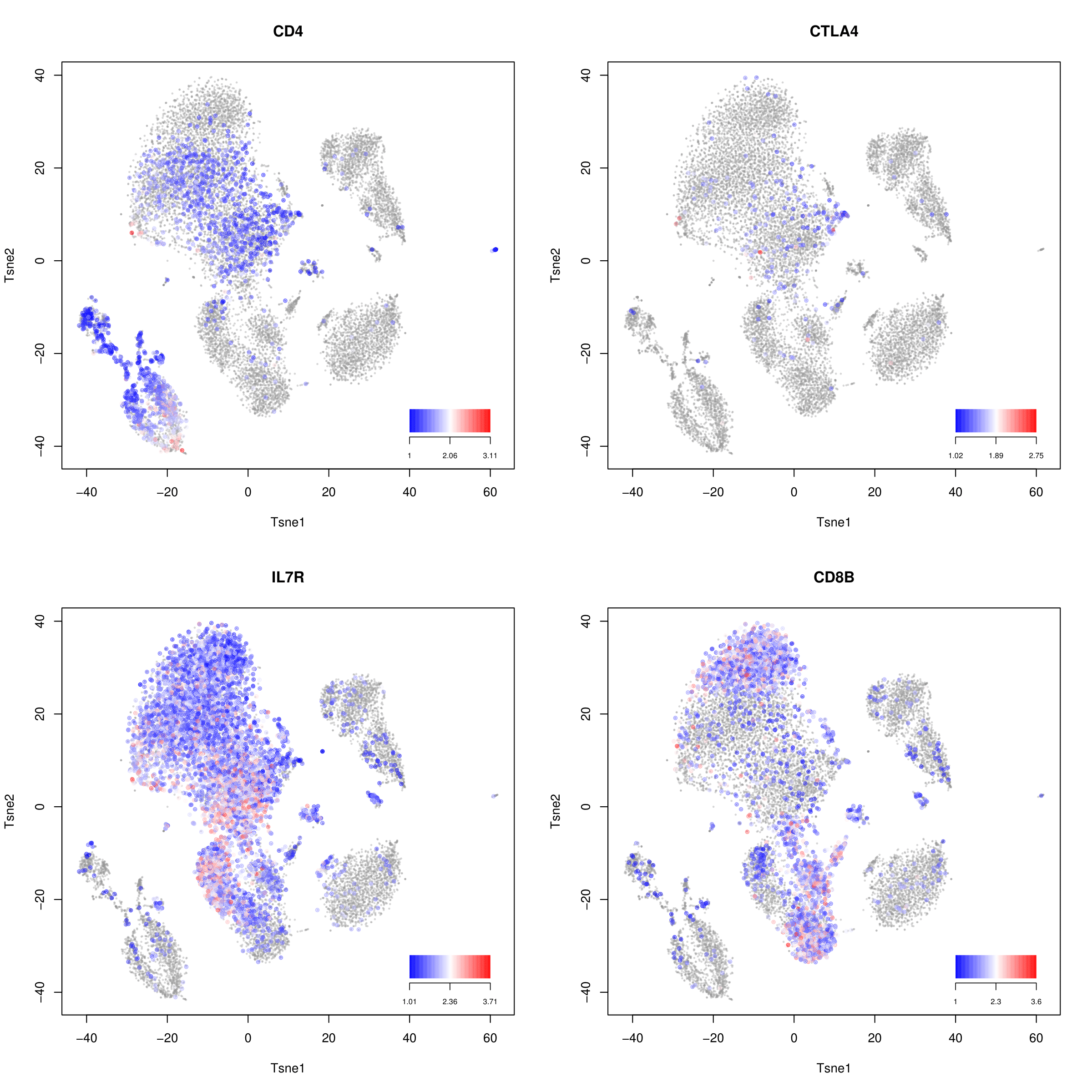
**

**
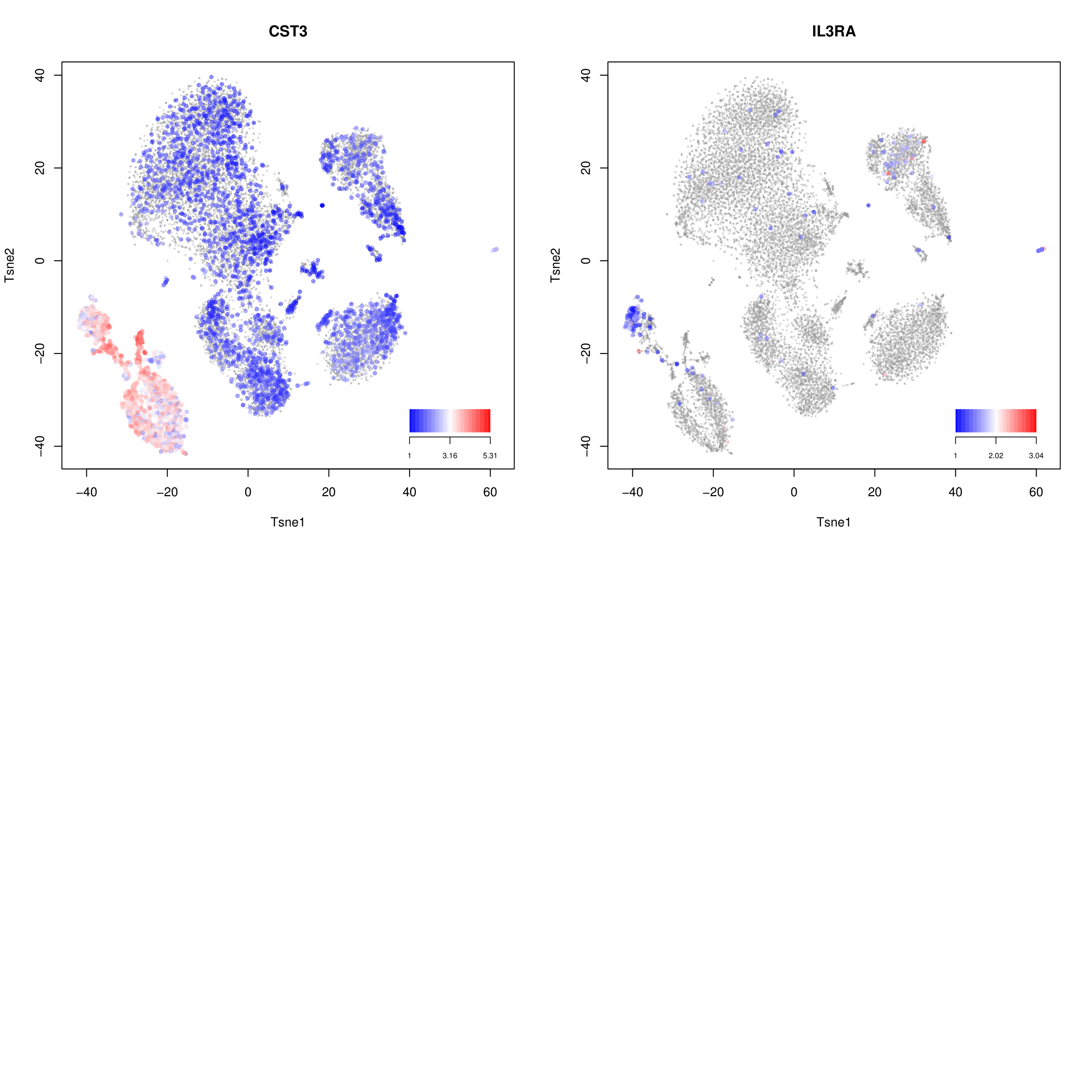
**

**Figure S3. Differential gene expression of 13 genes common induced by THC using qPCR in bulk PBMCs and scRNA-seq in six cell types. A. Heatmap of gene expression fold changes in bulk PBMCs and cell types; B. Concordance of fold changes between bulk PBMCs and CD8 cells.**

**
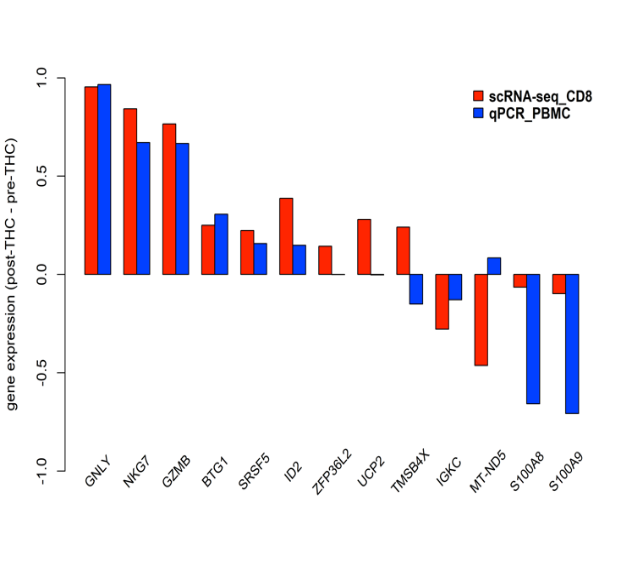

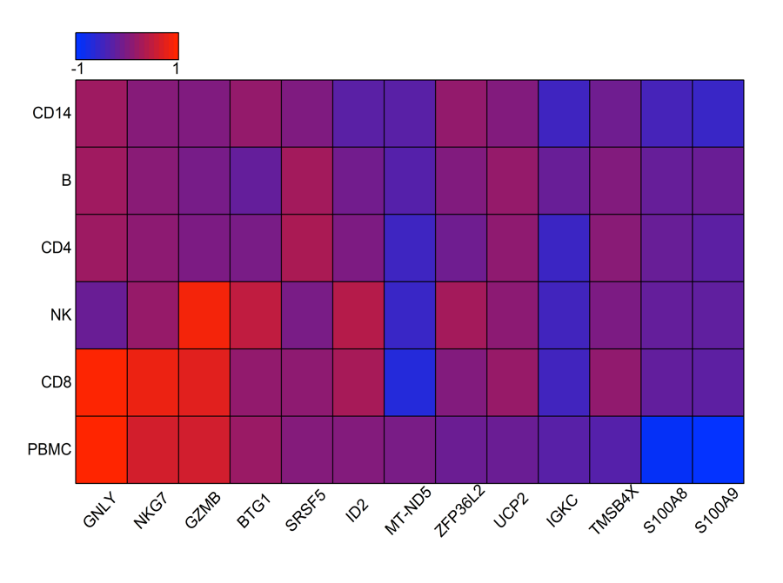
**

**Figure S4. Inferred mechanistic TNF gene networks involving in THC-induced gene expression. The expression of six THC-induced genes regulated by TNF pathway that includes 15 genes participating in signaling transduction**

**
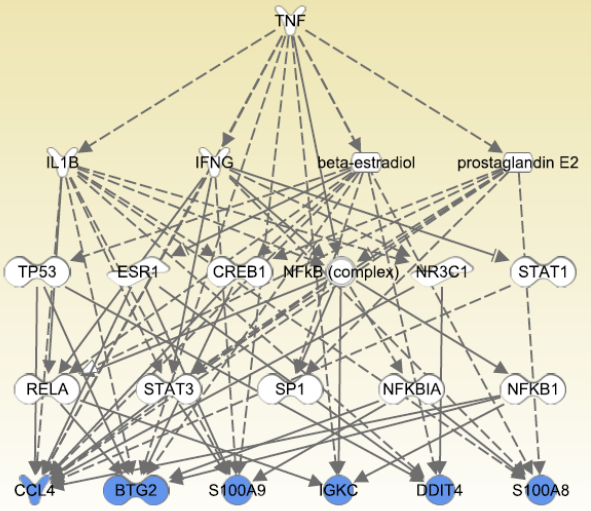
**

**Figure S5. Study design and procedure of THC infusion in human subjects.**

**
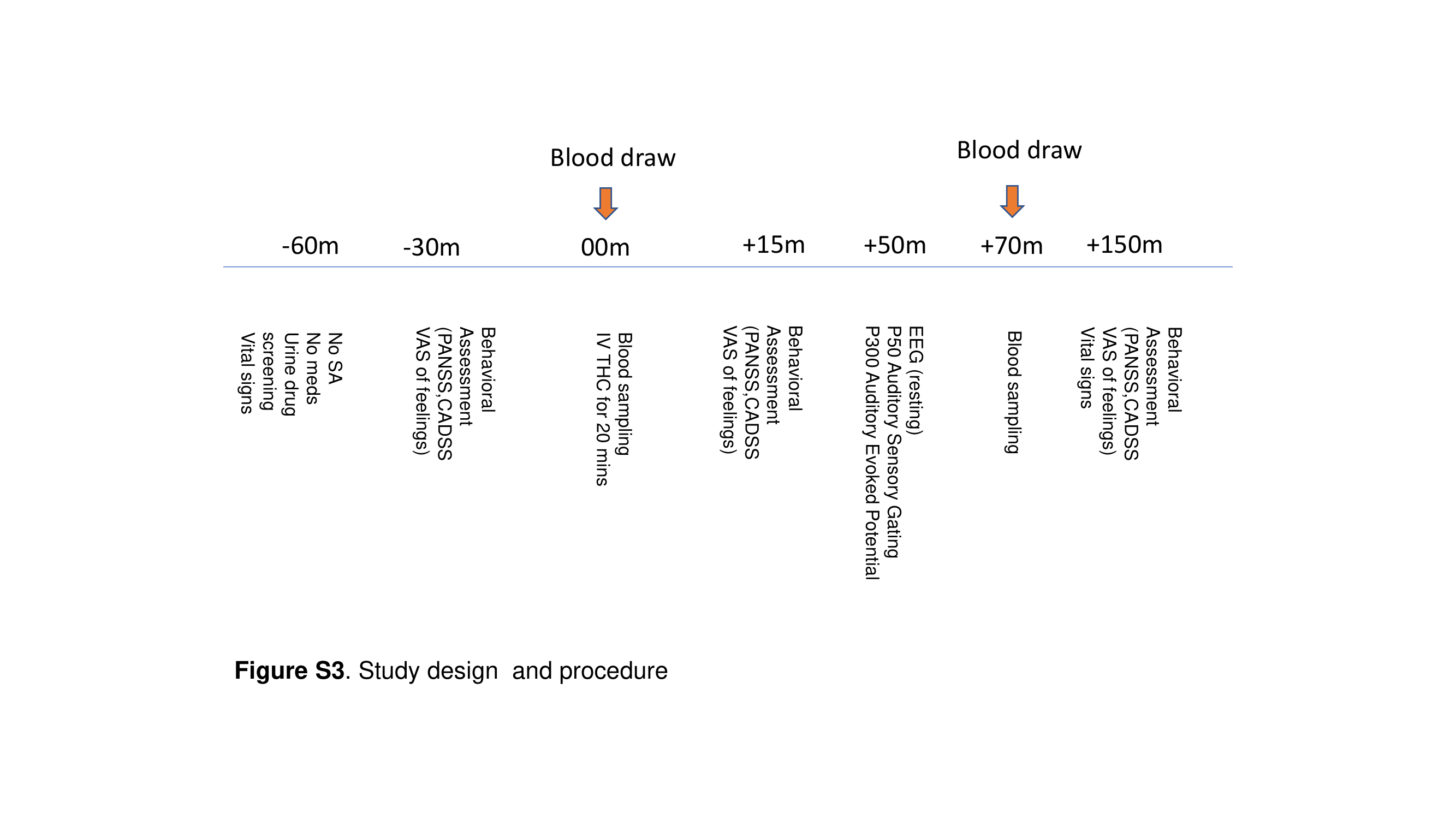
**

SA: Substance Abuse; PANSS: Positive and Negative Syndrome Scale; CADSS: Clinician Administered Dissociative States Scale; VAS: Visual Analog Scale; IV: Intravenous Injection; EEG: electroencephalogram.

**Supplementary Tables**

**Table S1.** Cell markers for cell type identification

**Table S2.** Mapping cell types using generalized linear modelling

**Table S3.** Proportion of cell numbers for each sample in a given cell type

**Table S4.** A summary of the number of differentially expressed genes between post- and pre-THC samples in each cell type

**Table S5.** Differentially expressed genes affected by THC in CD4+ T-cells

**Table S6.** Differentially expressed genes affected by THC in IL7RCD4+ T-cells

**Table S7.** Differentially expressed genes affected by THC in CD8+ T-cells

**Table S8.** Differentially expressed genes affected by THC in B cells

**Table S9.** Differentially expressed genes affected by THC in natural killer cells

**Table S10.** Differentially expressed genes affected by THC in CD14+ monocytes

**Table S11.** Differentially expressed genes affected by THC in FCGR3A monocytes

**Table S12.** log2 fold change of top 20 genes between post- and pre-THC infusion in each CD4+ T-cell clusters

**Table S13**. log2 fold change of top 20 genes between post- and pre-THC infusion in each CD8+ T-cell clusters

**Table S14.** log2 fold change of top 20 genes between post- and pre-THC infusion in each B cell clusters

**Table S15.** log2 fold change of top 20 genes between post- and pre-THC infusion in each CD14+ monocytes clusters

**Table S16.** Gene Ontology (GO) term enrichment for the co-expression network in CD4+ T-cells

**Table S17.** Gene Ontology (GO) term enrichment for the co-expression network in CD8+ T-cells

**Table S18.** Gene Ontology (GO) term enrichment for the co-expression network in B cells

**Table S19.** Gene Ontology (GO) term enrichment for the co-expression network in natural killer cells

**Table S20.** Gene Ontology (GO) term enrichment for the co-expression network in CD14+ monocytes

**Table S21.** Gene Ontology (GO) term enrichment for the co-expression network in FCGR3A monocytes

**Table S22.** A summary of the number of significant pathway in each cell type

**Table S23.** Significant pathways from KEGG in CD4+ T-cells

**Table S24.** Significant pathways from KEGG in IL7RCD4+ T-cells

**Table S25.** Significant pathways from KEGG in CD8+ T-cells

**Table S26.** Significant pathways from KEGG in B cells

**Table S27.** Significant pathways from KEGG in natural killer cells

**Table S28.** Significant pathways from KEGG in CD14+ monocytes

**Table S29.** Significant pathways from KEGG in FCGR3A monocytes

**Table S30.** Pre-THC gene co-expression between *CNR2* and other genes

**Table S31.** Post-THC gene co-expression between *CNR2* and other genes

**Table S32.** Physiological and psychological variables pre- and post-THC infusion for two participants

**Table S33** Top 30 bioinformatically inferred causal analysis for 294 THC-associated genes
